# Occurrence of Bullous Pemphigoid in a Longstanding Case of Rheumatoid Arthritis in an Indian Patient: A Rare Association

**DOI:** 10.7759/cureus.59226

**Published:** 2024-04-28

**Authors:** Kirti S Deo, Pooja Chaurasia, Yugal Sharma, Aayush Gupta, Nishtha Malik

**Affiliations:** 1 Dermatology, Venereology, and Leprosy, Dr. D. Y. Patil Medical College, Hospital & Research Centre, Dr. D. Y. Patil Vidyapeeth, Pune, IND

**Keywords:** systemic autoimmune disease, rare association, immunobullous disease, rheumatoid arthritis, bullous pemphigoid (bp)

## Abstract

Bullous pemphigoid is a subepidermal blistering disease that rarely involves the mucous membranes and possesses circulating antibodies against BP antigen II (BP180) and BP antigen I (BP230). Rheumatoid arthritis (RA) is a progressive inflammatory autoimmune disease that is characterized by joint inflammation and systemic involvement. The co-occurrence of RA, which is likewise linked to autoimmunity, with bullous pemphigoid may not be merely coincidental.

A 55-year-old female, a known case of RA for 25 years, presented to us with multiple pruritic vesiculobullous lesions. After a thorough clinical and laboratory assessment, she was diagnosed with bullous pemphigoid. This emphasizes the significance of the simultaneous occurrence of autoimmune disorders and the need for vigilant and timely identification.

## Introduction

Rheumatoid arthritis (RA), a chronic, persistent, progressive, symmetric autoimmune disorder, during its long natural course, may concurrently develop autoimmune disorders pertaining to several systems. Such dermatoses can range from the common ones, such as rheumatoid nodules, vasculitis, granulomatous dermatitis, and neutrophilic dermatoses, to the uncommon ones, namely panniculitis [1], erythema elevatum diutinum [2], alopecia universalis [3], psoriasis, and vesiculobullous diseases. Among the latter, bullous pemphigoid and its mucous membrane variant have been reported to be extremely rare; 32 reports to date include one from India, which documents the simultaneous presence of RA, bullous pemphigoid, and vitiligo [3, 4].

## Case presentation

**Figure 1:**
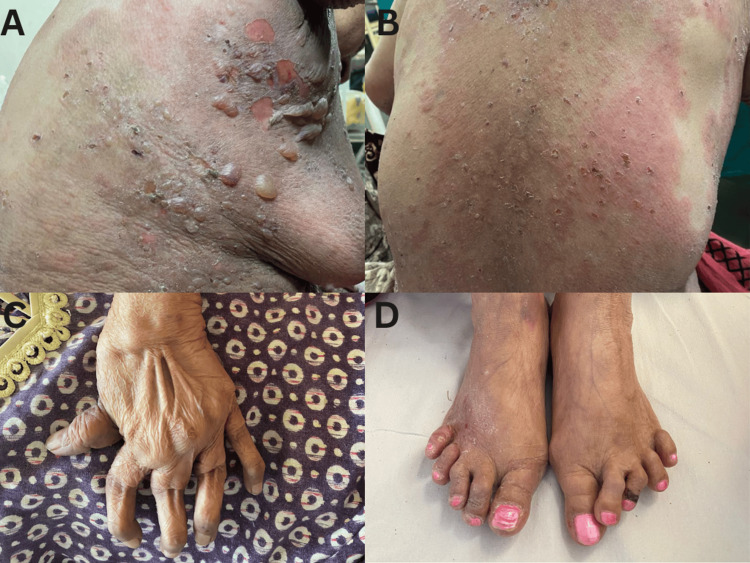
Cutaneous examination findings (A) Multiple fluid-filled vesicles and bullae, erosions, and crusts on the erythematous skin of the right infra-axillary region; (B) Extensive erythema, few vesicles, and numerous crusts on the back; (C) Hand changes denoting severe rheumatoid arthritis with subluxation of joints, swan neck deformity, and ulnar deviation of fingers; (D) Medial subluxation of the second and third toes of the left foot

Herein we report a rare co-occurrence of bullous pemphigoid in a 55-year-old female, a known case of RA for 25 years. She reported to us two months ago with a history of developing intensely itchy urticarial plaques progressively over limbs, trunks, and face, followed by the appearance over the plaques and intervening erythematous skin of multiple tense bullae, many erosions, and at places foul-smelling hemorrhagic crusts four weeks later (Figures 1A, 1B).

Her general physical examination was unremarkable except for the presence of pallor and multiple deformities of hands and feet characteristic of RA (Figures 1C, 1D), unfortunately, due to inadequate symptomatic management solely with nonsteroidal anti-inflammatory drugs. Dermatological tests revealed a negative bulla spread sign, as did Nikolsky tests. The intraoral examination was normal. The laboratory investigations (Table 1) revealed a hemoglobin level of 10.3 g%, leukocytosis amounting to 10,300/mm^3^, with 31% eosinophils, and an absolute eosinophilic count of 3,100/mm^3^ (normal range: 20-500). Elevated C-reactive protein (CRP) (4.1 mg/ml) and erythrocyte sedimentation rate (ESR) (42 mm first hour) underscored the presence of lingering systemic inflammation. Noteworthy serological findings included a markedly elevated RA factor of 386 units/mL (normal <15) and an anti-cyclic citrullinated peptide (anti-CCP) level of 57.4 units/mL (normal <20). A tabulation of laboratory values and their respective reference ranges has been included in Table 1.

**Table 1:** Laboratory parameters of the patient with reference range CRP: C-reactive protein; ESR: erythrocyte sedimentation rate; RA factor: rheumatoid arthritis factor; anti-CCP: anti-cyclic citrullinated peptide

Laboratory parameter	Values	Reference range
Hemoglobin	10.3 g%	12-15 g %
Leukocyte count	10,300/cubic mm	4,000-1,0000/cubic mm
Eosinophils	31 %	1-6%
Absolute eosinophilic count	3,100/cubic mm	20-500/cubic mm
CRP	4.1 mg/ml	<0.3 mg/ml
ESR	42 mm/hour	Upto 30 mm/hour
RA factor	386 units/ml	<15 units/ml
Anti-CCP	57.4 units/ml	<20 units/ml

**Figure 2:**
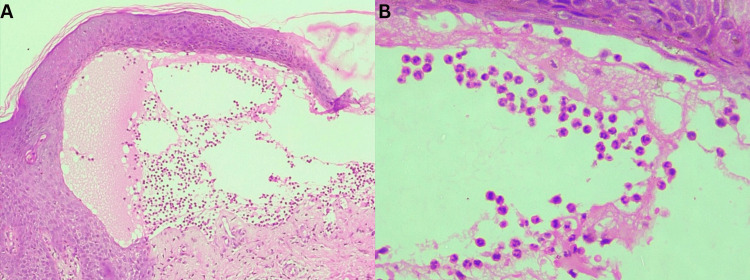
Histopathological findings (A) Microscopic photograph revealing subepidermal blister containing proteinaceous fluid and inflammatory infiltrate (H & E, 10x); (B) Microscopic photograph revealing infiltrate predominantly eosinophils with few neutrophils within the subepidermal cleavage (H & E, 40x).

Histopathological examination of the skin lesions, revealing subepidermal blisters predominantly populated by eosinophils (Figures 2A, 2B), was consistent with our impression of bullous pemphigoid. Unfortunately, due to financial constraints, immunofluorescence studies could not be performed. The patient exhibited substantial relief within a relatively short span of six weeks following therapy with 30 mg prednisolone, 100 mg dapsone, and 25 mg doxepin per day.

## Discussion

The most widespread autoimmune bullous disorder, bullous pemphigoid, is characterized by the development of autoantibodies that target hemidesmosomal proteins found in the skin and mucous membranes, specifically dystonin e, now referred to as BPAg1 e, and collagen XVII. This condition primarily affects the elderly population and has shown a gradual increase in incidence, correlating with significant levels of morbidity and mortality.

Clinically, bullous pemphigoid manifests with a profoundly itchy rash accompanied by widespread tense subepidermal bullae, which are located on the trunk or extremities and arise from normal or erythematous skin. Identifying bullous pemphigoid can be challenging, particularly when presentations deviate from the typical pattern. Diagnosis relies on a comprehensive assessment integrating clinical, histological, immunopathological, and serological findings. Topical and/or systemic glucocorticoids are the mainstay of treatment while steroid-sparing adjuncts and antibiotics are good alternative treatments [5].

Additional treatments that can be supplemented with corticosteroid regimens for generalized bullous pemphigoid encompass immunosuppressive therapies like azathioprine, mycophenolate mofetil, methotrexate, cyclophosphamide, cyclosporine, chlorambucil, and leflunomide. These treatments are also applicable to RA [6]. For generalized bullous pemphigoid unresponsive to initial treatments, alternative or supplementary therapies involve immunomodulators or biologics such as intravenous immunoglobulin (IVIg), plasmapheresis, rituximab, dupilumab, and omalizumab [7].

In contrast to the solidity of the assumption of the loss of dermal-epidermal adhesion due to autoantibody binding, more precise aspects of the upstream and downstream mechanisms underlying the development of bullous pemphigoid are still heavily conjectured. An imbalance between autoreactive T helper (Th) cells and T regulatory cells has been identified as the primary pathogenic mechanism eliciting the autoimmune response in patients with bullous pemphigoid disease.

Nevertheless, the exact impact of signaling pathways that enhance B cell activation, like Toll-like receptor activation, and other inflammatory mechanisms that cause blister formation, such as induction of the Th17 axis and initiation of the coagulation cascade, is still being discussed and disputed [8].

Rheumatoid arthritis is a progressive inflammatory autoimmune condition affecting both joints and the body as a whole. While its exact cause remains unknown, a combination of genetic and environmental factors is believed to contribute to its development. B cells, T cells, and the coordinated action of pro-inflammatory cytokines play pivotal roles in the pathophysiology of RA.

The differentiation of naïve T cells into Th17 (TH17) cells leads to the production of interleukin (IL)-17, a potent cytokine that promotes inflammation in the synovial tissue. B cells contribute to the disease process by presenting antigens and producing autoantibodies and cytokines. Synovitis is often initiated at the synovial membrane, where the infiltration and/or local activation of mononuclear cells and the development of new blood vessels lead to joint injury.

CD4+ T cells, once activated by antigens, enhance the immune response by stimulating other immune cells, such as chondrocytes, synovial fibroblasts, and osteoclasts. Inflammation occurs in the synovium as a result of the release of cytokines, specifically IL-6, tumor necrosis factor-alpha (TNF-α), and IL-1 [9]. 

The infiltration of eosinophils into the skin is seen as an important and early step in the formation of blistering lesions in bullous pemphigoid. The count of peripheral eosinophils has been demonstrated to provide a more precise indication of disease activity compared to levels of anti-BP180 [10].

The coexistence of bullous pemphigoid and RA, both autoimmune diseases, raises intriguing possibilities regarding their interconnectedness, suggesting a potential shared underlying mechanism; one plausible hypothesis is the occurrence of an immune reaction between antigens within the basement membrane zone (BMZ) and those present in the synovium.

Alternatively, the inflammatory milieu associated with synovial inflammation in RA could act as a catalyst, unveiling specific antigens (BPAg1 and BPAg2) in the BMZ of the skin. Consequently, rather than a mere coincidental convergence, bullous pemphigoid and RA might be intricately linked as associated diseases, sharing a common immunological landscape [11]. This proposition gains support from previous reports of cases in the literature of pemphigoid, encompassing its cicatricial form, in conjunction with RA [12].

## Conclusions

Hence, in patients with RA, apart from clinical vigilance and prolonged monitoring for seeking their concurrence, autoimmune conditions like bullous pemphigoid also need to be reported. Timely and thorough investigations of immunological aspects therefore become imperative, especially when a pre-existing autoimmune disorder is already diagnosed. 

This vigilance may lead to the potential emergence of additional autoimmune diseases and scrutiny of the intricacies of the involvement of the immune system, shedding light on their pathogenesis and hopefully paving the way for more targeted diagnostic and therapeutic approaches.
